# The Nexø Method—Clinical Evidence for the Paradigm Shift in Caries Management for Children and Adolescents in Denmark Being Cost-Effective

**DOI:** 10.3390/children13030432

**Published:** 2026-03-22

**Authors:** Kim Rud Ekstrand, Mauri Erik Christian Christiansen

**Affiliations:** 1 Department of Odontology, Faculty of Health and Medical Sciences, University of Copenhagen, 2200 Copenhagen, Denmark; 2 Former Nexø Public Dental Health Service, Municipality of Nexø, 3730 Nexø, Denmark; mauri@mail.dk

**Keywords:** child, adolescent, dental caries, prevention of caries, fear, anxiety

## Abstract

**Highlights:**

**What are the main findings?**

• The Nexø method—a Danish non-operative caries-control program based on three principles, dosed at individually assessed recalls according to diagnosis and risk assessment—was very effective in controlling caries disease in children aged between 0 and 18 yrs of age.
• It is highly likely that the Nexø method promoted a marked reduction in anxiety-provoking dental treatments in Nexø, as nearly 80% of the 15-year-old population in Nexø had a DMFS = 0, compared to 40% at the Danish national level.

**What are the implications of the main findings?**

The overall objective with the Nexø method was to keep teeth healthy, which requires due diligence.

• For primary dentition, non-operative interventions should start at the time of eruption of the first primary teeth, which occurs when the child reaches the age of 8 months.
• For permanent dentition, non-operative interventions should start at the time of eruption of the permanent first molar teeth, which happens at around the age of 5–7 years old.

**Abstract:**

**Introduction**: In the period from the 1960s to the new millennium, dental care for children in Denmark focused mostly on drilling and filling lesions once they appeared. This often led to repeated operative treatments, not to mention the trauma related to the many anxiety-provoking dental treatments undergone by these children. **Aim**: It is cost-effective to document, by means of clinical data over a 25-year period, that the paradigm shift from operative management of caries to a more non-operative approach. **Method**: The name of the program is the Nexø method, which was developed in 1987 in the Municipality of Nexø (one of 275 municipalities) in Denmark. The method was operationalized into a structured approach based on three principles, dosed at individually assessed recalls according to diagnosis and risk assessment. The risk assessment was based on four risk criteria, each divided into a “good” (1 point) or “bad” (2 points) situation, which were eventually used to assess the length of the interval between visits to the clinic. **Outcomes**: National Danish oral health data (SCOR) and oral health data from the Nexø municipality from 1985 to 2005 were analyzed, descriptively as well as statistically (Welch’s *t*-test, 95%CI and Cohen’s d), to compare the caries experience on a national level with data from Nexø in terms of mean defs/DMFS and percentage of 5-, 15-, and 18-year-olds with a defs/DMFS = 0. **Results**: The mean defs/DMFS or percentage of children with a defs = 0/DMFS = 0 in Nexø was, prior to the implementation of the Nexø method (before 1987), at the national level or worse. The mean defs/DMFS dropped significantly (*p*-values < 0.0001) from 1990 onwards in Nexø in 5-, 15- and 18-year-olds compared to national data. The DMFS = 0 among 15-yr-olds in Nexø reached 80% in 2005 compared with 40% nationally in the same year. For 15-yr-olds in 2005, the effect size expressed by Cohen’s d = −0.43, indicating a moderate effect of the Nexø method. The mean number of sealed surfaces in 2003 was 3.1 (1SD = 1.6) in 39 reporting municipalities, and for Nexø the mean value was 2.8 surfaces. The cost (price/child/year) was under control over the years. **Conclusions**: The Nexø Method shows the clinical evidence that the paradigm shift to prevention of the disease process, rather than operative intervention, is cost effective, even with a rather limited use of sealants. A reduction in fear and anxiety-provoking dental treatments in Nexø compared to most other municipalities in Denmark is expected.

## 1. Introduction

The caries situation was very serious in Denmark in the 1950s, 1960s and 1970s [1]. Due to that fact some municipalities independently established child dental health services for the schoolchildren in their municipality. The government was against this unequal practice in Denmark, which was why the Danish Parliament passed a law in 1971 which stated that from 1972, all municipalities (*n* = 275) should establish free oral health care in public clinics, initially covering every pupil between 7 and 15 years of age. The scheme was called the Municipal Child Oral Health Care Scheme at the time (MCOHCS), as the individual municipality was made responsible for implementing and running the dental service for the children/adolescents living in the municipality. All schoolchildren in Denmark were to be under the scheme by 1980. From 1981 to 1985, the scheme was extended to include 0- to 6-yr-olds, and in 1986 and 1987, 16- and 17-yr-olds were also included in the scheme. In 2021, it was decided that 19- to 21-yr-olds should also join the scheme.

From its establishment in 1972, the scheme covered:

1. Regular dental examinations.
2. General and individual preventive measures.
3. Dental treatment, including orthodontics, necessary to keep the mouth and teeth in good working order.
4. The service should be outreaching; thus, the service was responsible for calling the children to the clinic. If the child did not turn up, recalls were made, in accordance with the law, until the child finally turned up at the clinic.

The chief dental officer appointed by the political administration in each municipality had and still has the overall responsibility for executing the service and thus had huge freedom to plan the service.

A registration system for the child’s oral status was established at the start of the 1970s by Helm and coworkers [2] and was, in Danish, abbreviated to SCOR. Locally, each child had their dental health recorded once a year on a special form. Copies of the forms were then sent to the Danish Health Authority Statistics, which treated the data and expressed the oral health data yearly on national, regional, and municipality levels in reports, as well as storing the data in the Danish Health Authority’s data bank [2]. Since 1993, the registration of oral health status has been only obligatory for age cohorts of 5-, 7-, 12-, and 15-yr-olds, but if it was the wish of the local chief dental officer, they could decide to record the children at any ages and get the outcome cohort data from any age from the Danish Health Authority Statistics.

In the period from the 1970s to the start of the new millennium, dental caries was mainly perceived by the MCOHCS as a disease requiring operative treatment once lesions had been detected. For decades, the clinical standard was the “drill and fill” paradigm, where success was measured by the number of restorations placed rather than the maintaining of natural tooth structure. Prevention was carried out as part of general programs in kindergartens and schools, but the two dental schools in Denmark argued for a more individualized approach, to control for caries related to the individual needs of the individual child. This required that the personnel in the MCOHCS undertook more risk-related work, with individual assessed recall intervals instead of school class examinations every 6 months, where all pupils in a class had their examination on the same day.

The aim of this paper is to document, by means of clinical data over a 25-year period, that the paradigm shift from operative management of caries to a more non-operative approach was cost effective. The program was named the Nexø method [3,4]. The program was launched in two timepoints: (1) for the permanent dentition implemented in 1988, and (2) for the primary dentition in 1990.

## 2. Devising the Nexø Method

In the late 20th century, dental caries was recognized as a localized disease, caused by local evolution of microbial biofilms in plaque stagnation areas (PSA) where mechanical wear on the tooth surface was limited. This opened the possibility for programming non-operative caries management, inspired by the work of the late Professor Thylstrup and Professor Fejerskov, both from Denmark [5,6,7,8,9]. This thinking culminated in the development of the Nexø method—a non-operative caries controlling program executed in the Municipality of Nexø at Bornholm in the late 1980s. As the Nexø method was a risk-based program [10,11], presumably the first longitudinal approach in the MCOHCS, it represents a radical departure from the traditional operative model. The shift can be described, in Kuhn’s terms, as a true paradigm shift [12].

### The Content of the Nexø Method

The treatment program was operationalized into a structured approach [10,11] based on 3 principles, dosed at individually assessed recalls, according to diagnosis and risk assessment:

1. Parents and children were taught the local nature of the caries disease.

2. Intensive training in home treatment (tooth brushing). Schoolchildren had their teeth stained with a disclosing agent at every visit to the clinic.

3. Professional plaque removal, drying, and diagnostics. In the case of caries progression, specific education and training in plaque removal was implemented, followed by topical application of fluoride if initial caries were detected. Finally, individualized risk assessment was carried out to decide the length of the interval to the next visit at the clinic.

Successful implementation of the method required that the dental staff (dentists, dental hygienists and auxiliary personnel) possessed thorough knowledge and understanding of the localized nature of dental caries [5] according to the following principles.

Caries is caused by bacteria present in the oral cavity. Changes in conditions of growth leading to accumulations of undisturbed bacteria on teeth initiate decalcification due to excessive acid production in the plaque. Mechanical forces, such as contact between teeth, between tongue and teeth, chewing, tooth brushing, etc., prevent caries progression due to disturbance of plaque, whereas fluoride may only decelerate the progression process. Carbohydrates, on the other hand, may accelerate caries progression rate underneath undisturbed plaque [5,6,7,8,9].

Therefore, caries control requires disturbance of bacteria accumulations to prevent acid production from reaching a level resulting in net loss of mineral from the tooth. A general recommendation to parents/children was to brush teeth before breakfast and before bedtime using fluoridated toothpaste, stressing the importance of quality of the plaque removal.

Further, the criteria in Table 1 for when to recall were established and used for all patients at the clinic.

The 4 risk criteria used were each divided into a “good” (1 point) or “bad” (2 point) situation. The interval length was then determined from the sum of points: one month (8 points), 2 months (7 points), 3 months (6 points), 4 months (5 points) and 6 months or more (4 points). As seen, the eruption stage of the permanent molars was considered to be a risk factor [7] in this method, and was categorized as full occlusion being a good condition, and partial occlusion a bad condition.

**Figure 1:**
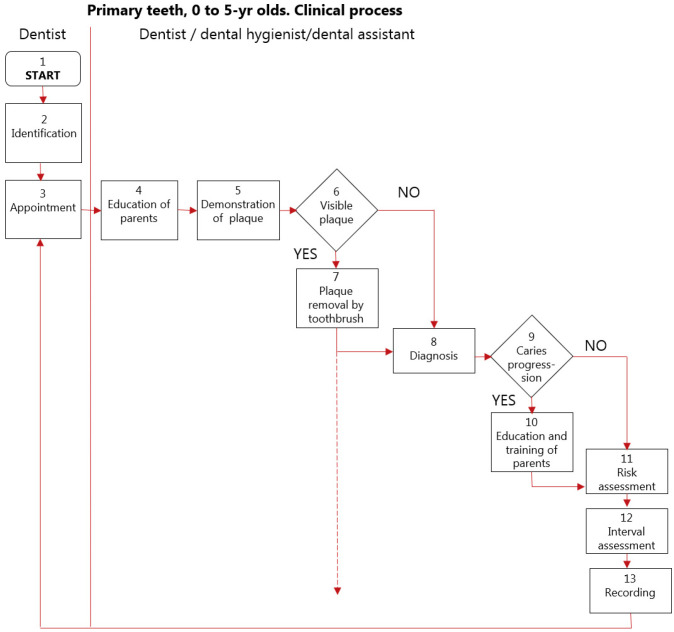
Flowchart showing the clinical process in primary dentition from the age of 8 months old to when the permanent first molars start to erupt. Red arrows indicate progress one event to another. The dotted line indicates that the child is too small for the diagnostic process to take place, but the risk assessment is still performed.

Figure 1 is a flowchart illustrating, as an example, the clinical process used in the Nexø method. Figure 1 shows the dental stages from when the primary teeth erupt to the permanent first molar teeth erupt. From the age of about 6 to the age of 18, the teeth were discolored to identify the cooperation of the parents/the child/young adult themselves. Boxes 4–7 focus on controlling one of the necessary factors for caries to develop: dental plaque [5,6]. The caries diagnostic process is performed on clean teeth (box 8). If no active caries is diagnosed, risk assessment using the criteria in Table 1 is performed. If active initial caries is diagnosed, it is shown to the parents to visualize where better plaque control should be performed, fluoride applications are applied, and a new risk assessed interval is planned (box 10,11). If caries still progresses on the next visit, revisits with short intervals can continue; eventually, but very rarely, operative interventions are needed. A detailed description of the clinical process on permanent teeth used in the Nexø method is given in [10].

## 3. SCOR Data

In the SCOR system, caries and its sequela were scored as follows on the forms: Blank = sound surface, 0 = primary initial lesion, 1 = primary manifest lesion, 2 = secondary manifest lesion, 4 = restored due to caries, 5 = endodontically treated due to caries, 6 = extracted due to caries, 8 = sealed surface and 9 = arrested lesion. In the defs/DMFS index used for SCOR data, only scores 1, 2, 4, 5 and 6 were included.

The SCOR system has changed slightly over the >50 years it has existed but has in general been said to be easy to use in clinical settings, operating with only two stages of caries, initial and manifest (where manifestnearly always leads to operative intervention). As the personnel are repeatedly calibrated in its use, the data observed regarding caries, the SCOR data, is reliable according to Skeie et al. 2014 [13].

## 4. Statistical Considerations

National SCOR data provides the mean defs/DMFS values, the standard deviation (SD) on the mean, and the number of children recorded. Similar data was available for the individual municipalities. The national birth cohorts in Denmark in the end of the last century were around 65,000 children. The number of recorded children at national level for 5- and 15-yr-olds was around 55,000 each year in the period from 1985 to 2005. Thus, not all who should have had their oral health status recorded were recorded. Even so, the Danish Health Authority Statistics reported those data for data at the national level. For the 18-yr-olds, the number recorded was about 25,000, as it was not compulsory to record the oral health status of 18-yr olds. On the other hand, at the age of 18, young adults untill 2021, had to leave the free dental health service, so many municipalities were interested in recording what they had achieved concerning oral health during an 18-year period.

The size of the birth cohorts in Nexø for those years was around 100 children, and >95% of those allocated to the clinic had their dental health recorded every year.

Welch’s *t*-test [14] was used to test for statistical differences between the outcomes in Nexø (test) versus national data (control). A 95% confidence interval was presented for all 3 age groups and Cohen’s d (measure the effect size) was calculated (Excell) for the 15-yr-olds in the year 2005.

## 5. Results

### Outcomes for the Nexø Method (Caries Prevalence)

**Figure 2:**
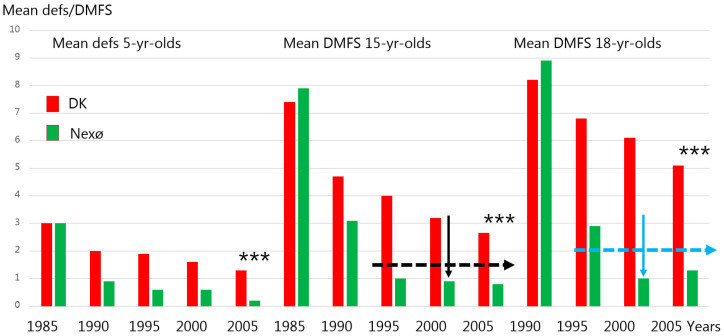
Mean defs or DMFS indexes of 5-, 15- and 18-yr-olds in Nexø and at national level in Denmark in 1985 to 2005. The dotted lines indicate the targets which should be reached in Nexø from 2000 and ahead.

**Figure 3:**
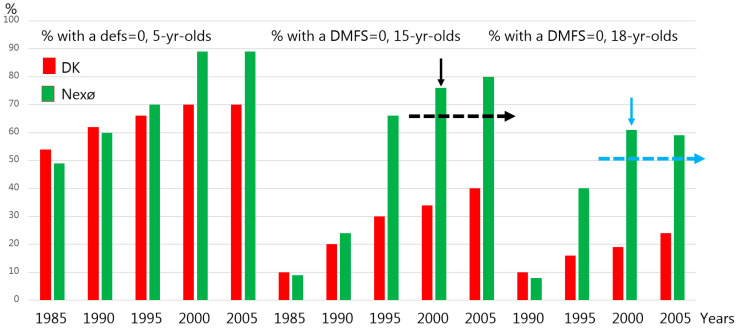
Prevalence in % of children aged 5-, 15- and 18-yrs-old with a defs = 0 and DMFS = 0 in Nexø and at national level in Denmark in 1985 to 2005. The dotted lines indicate the targets which should be reached in Nexø from 2000 and ahead.

The outcomes in terms of mean defs/DMFS or percentage of children with a defs = 0/DMFS = 0, reported yearly by the Danish Health Authority Statistics, was, up to the mid-1980s in Nexø, at the national level or worse (Figure 2 and Figure 3; see the columns for the year 1985).

In 1988, the chief dental officer in Nexø suggested the following target outcomes to be achieved in the year 2000 (Table 2).

These targets were met in around 2000, as seen in Figure 2, where the mean DMFS in 15-yr-olds in Nexø municipal area was 0.88 (black arrow). For the 18-yr-olds, the mean DMFS = 1.03 (blue arrow). The stars in Figure 2 on all three age groups in 2005 indicate that the achieved results in Nexø in terms of mean defs/DMFS were significantly lower (*p* < 0.0001) than at the national level. The difference in means for the 15-yr-olds in Nexø versus the national level was 0.75–2.65 = −1.9 and the 95% CI values were −2.23 to −1.57 (t = −11.51); For the 18-yr-olds, the mean difference was 1.33–5.15 = −3.82 and the 95% CI values were −4.44 to −3.19 (t = −12.3). Cohen’s d for the 15-yr-olds in 2005 was −0.43.

With respect to the DMFS = 0 outcome, in 2000, 75%% of the 15-yr-olds in Nexø (Figure 3, black arrow) and 60% of the 18-yr-olds (Figure 3, blue arrow) were classified in this category. Similarly, data at the national level were 33% and 19%, respectively.

Representative data from a total of 39 municipalities in Denmark [15] showed that the mean number of sealed surfaces on 15-yr-olds in 2003 varied between 0.26 in one municipality and 6.0 in another municipality, with a mean of 3.1 (1SD = 1.6) for the 39 municipalities. The mean number of sealed surfaces on 15-yr-olds in Nexø that year was 2.8 sealed surfaces.

Concerning caries in primary dentition, it appears that there was a similar tendency to that observed for permanent dentition. Before the Nexø method, the level of caries was the same or slightly worse than at the national level, while shortly after implementation of the Nexø method in primary dentition, the caries experience became significantly better than that at the national level. Thus, mean defs in 2005 was 0.19 in Nexø versus 1.36 (*p* < 0.001) at the national level. The mean difference was then −1.17 and the 95% CI values were −1.31 to −1.03) (t = −17.1) in 2005 (Figure 2). The % 5-yr-olds with a defs = 0 was close to 90% in Nexø versus 70% at the national level.

## 6. Discussion

The annual SCOR-data has proved extremely valuable for the evaluation of preventive initiatives in the MCOHCS in Denmark over the years. As described in the Results section, the outcomes were very favorable in favor of Nexø compared to the national level. For example, the 95%CI for the 15-yr-olds in 2005 was narrow ((−2.23 to −1.57) = −0.66) seen in relation to the difference between the means (−1.9), indicating a precise estimate of the effect. Further, the value 0 was not in the interval and far from the lower CI value on −1.57, confirming that the *p* value was <0.0001. We chose to express the level of effect size of the Nexø method using only the 15-yr-olds in 2005, as the number of 15-yr-olds in Nexø that year was relatively high, at 146. Similar numbers of 5- and 18-yr-olds that year were 77 and 43, respectively. The effect size using the mean DMFS on the 15-yr-olds (0.75) was −0.43 and can be graduated close to a moderate effect size for the Nexø method according to Cohen’s classification [16]. So, the effect of the Nexø method is not only statistically significant, but also clinically relevant.

We are aware of the ecological nature of our data, which called for investigation of the influence of confounding factors. This was tested in studies in 1999 and 2004 [10,11]. A total of eight background variables were accounted for during the analyses, and involved data from >93% of the municipalities. The statistical regression analyses disclosed that Nexø was considered an outlier in both studies. This means that the eight background variables, including inter-municipality differences in the fluoride concentration in the water supply, the mother’s level of education, or personal income, could not explain the results obtained with the Nexø method.

Another tested background was the price/child per year. The intermunicipal ranges in cost means of the involved years were between 762 and 2785 DKK; first quartile: 1230, median quartile: 1303, third quartile: 1420. The average cost per child per year in Nexø was placed in the median quartile [11]. At that time, 1 US$ equaled approx. 7 DKK.

MCOHCS still exists, but its workload has changed radically, for many reasons. The most important factor was the significant rise in the number of children and adolescents with a different cultural background from that of the traditional Danish cultural background. Further, the MCOHCS was also obligated to take care of the dental care of elderly vulnerable persons, starting from the mid-1990s. It is known that MCOHCS in other municipalities have included parts of the Nexø method in the last three decades [11] in their effort to control the caries disease in their individual municipalities. That the eruption period of permanent molars should be seen as a caries risk factor is well considered all over MCOHCS. National SCOR data support that; for example, the percentage of 15-yr-olds with a DMFS = 0 during the last decade approached the same level at the national level of that achieved in Nexø in 2005 [17].

In 2007, the 275 Danish municipalities were reduced to 98. As part of this reform, Nexø municipality was united with four neighboring municipalities to create one big municipality named Bornholm. Specific SCOR data were available from Nexø until 2007. Since then, the Nexø data has been part of the Bornholm data. Since 2006, the SCOR data from Bornholm has been better than (2006 to 2018) or near to the national level (2019 to 2023).

There are several key elements which justify our consideration of the results obtained with the Nexø method as evidence that paradigm shifts in management of caries from an operative to a non-operative approach took place and were cost effective.

The program was devised to maintain teeth soundness, which was related to the fact that in Nexø in 1985, only 9% of the 15-yr-olds had a DMFS = 0 (Figure 3), while the mean number of DMFS was close to 8 (Figure 2). Similar data was seen at the national level. Thus, the clinical program devised celebrates the principle of due diligence, meaning the initiation of caries control initiatives before caries develop. A good example of this is to call in children to the clinic at the time when the primary teeth start to erupt (around 8 months of age) (Figure 1) and when their permanent molar teeth start to erupt. The long-lasting eruption period of permanent molar teeth [18] is considered as a true risk factor, according to Carvalho et al. [7]. Equally important is that a caries risk assessment tool was devised (Table 1) and used, and based on that an interval between recalls to the clinic could be decided according to the caries risk of the child. All this was totally new in the MCOHCS in Denmark in the late 1980s.

Further local fluoride applications (2% NaF solution) were used in Nexø but only based on an active caries diagnosis (initial lesions), as suggested by Fejerskov et al. in the early 1980s [19]. In Nexø, sealing was not considered a routine preventive measure but was used only selectively for children with documented caries risk, using the criteria in Table 1. The philosophy of restrictive use of fissure-sealed surfaces [10,11] is reflected in the mean number of sealed surfaces being 2.8 for 15-yr-olds in 2003 [15], which was just below the mean of 3.06 found in the other municipalities. Because SCOR registration of sealed surfaces was/is voluntary and inconsistently recorded, there is no robust national data set to compare directly with Nexø, other than the paper by Ekstrand et al. [15]. Use of fissure sealants opens an important international discussion. Should prophylactic sealing be considered prevention, or is it in fact a “primitive” restoration? The Nexø experience demonstrates that a population can maintain high levels of sound tooth surfaces or arrested lesions with minimal reliance on sealants.

Among many other scientists [20], Thylstrup and Fejerskov [5,6,9], two Danish scientists, were central figures in describing the fundamental change in the understanding of caries from being classified as an infectious disease to being understood as a biofilm-mediated, diet-dependent, multifactorial disease [21]. This shift in paradigm changed the focus from the drill, fill and bill concept to the prevention of the disease process during the period of the 1990s and at the beginning of the 2000s. The Nexø method started in the late 1980s, and the data presented in this paper give the clinical evidence for that the paradigm: prevention of the disease process, rather than operative intervention, stands as it can control for caries development. The Nexø method can be seen as an individualized population strategy within caries control; all children/adolescents require the same type of nonoperative interventions, but some need the interventions more often than others, according to individual needs.

Finally, and even though we have no strong data to support it, it is tempting to speculate on whether the Nexø method also promoted a marked reduction in fear and anxiety provoked by dental treatments compared to most other municipalities in Denmark. When preschool-, school children and adolescents are exposed to operative intervention due to caries in Denmark, both in the past and today, the vast majority receive local anesthesia before the operative intervention. No doubt that local anesthesia provokes anxiety [22]. Logically, when only 20% of the 15-yr-olds in Nexø but 60% of the 15-yr-olds at the national level (Figure 3) received one or more operative interventions, the clinical processes in the Nexø method likely led to a reduction in anxiety-provoking dental treatments compared to the majority of other municipalities in Denmark. This contrasts with what the literature tells us: a significant global prevalence of children suffer from dental fear and anxiety [23]. Hence, the authors strongly recommend that dentists and dental hygienists all over the world test whether all or at least some of the interventions from the Nexø method can be used in their surroundings. Today, by using tele-dentistry it is possible to teach parents at home when and how to perform toothbrushing for their child, at the time of eruption of the first primary tooth, or the time of eruption of the permanent molar teeth, highlighting the principle of due diligence.

### For the Future

Based on the experience of the Nexø method, the authors recommend the following to control for caries in children and adolescents in the future, whether dentistry is performed at public clinics funded by the government or under private enterprise conditions:

• Start the caries controlling initiatives before caries can develop, by explaining to the parents how caries can be controlled.
• Focus on toothbrushing using fluoridated toothpaste, twice a day [24,25].
• The amount of fluoridated toothpaste should be limited to the size of the child’s little fingernail per day up to the age of 8.
• Focus on quality of plaque removal during toothbrushing or flossing.
• Focus on restricted intake of fermentable carbohydrates.
• Focus on risk assessment, for example as suggested in Table 1.
• Use local application of fluoride varnish every 3–4 months if the caries risk is moderate or high or there are diagnosed initial active lesions [26].
• Use sealants on lesions which progress, even if efforts have been made to control the progression [27].
• Use BW on nearly all patients in the following dental ages:
  ○ 1½ year after contact has been established between primary molar teeth.
  ○ 1½ year after contact has been established between primary 2nd molar and permanent 1st molar teeth.
  ○ 2 years after approximal contact has been established between permanent 1st and 2nd permanent molar teeth.
• Document when a tooth surface is carious, sealed, restored, or extracted due to caries

## 7. Conclusions

The Nexø method was introduced in Denmark in the late 1980s, coinciding with a gradual paradigm shift from operative management of caries toward a non-operative, preventive approach. The data presented in this paper provide clinical evidence that focusing on the prevention of the disease process, rather than on operative intervention, is cost-effective. Moreover, the Nexø method likely contributed to a reduction in fear- and anxiety-provoking dental treatments.

## Figures and Tables

**Table 1:** Risk criteria used for the interval planning.

		Permanent Teeth	Primary Teeth
		POINT	POINT
Cooperation	Inadequate	4	4
	Good	2	2
Caries activity	Yes	2	4
	No	1	2
Eruption of permanent molar teeth	Partly	2	
	In full occlusion	1	

**Table 2:** Target outcomes for Nexø in the year 2000.

	Mean DMFS	% Children/Adolescents with a DMFS = 0
15-yr-olds in 2000	<1.5	>2/3
18-yr-olds in 2000	<2.0	>50%

## Data Availability

The data presented in this study are openly available in https://sundhedsdatastyrelsen.dk/data-og-registre/nationale-sundhedsregistre/boerne-og-ungdomstandpleje (accessed on 14 March 2026).
